# *catena*-Poly[manganese(II)-(μ_2_-3,5-di-2-pyridyl-1,2,4-triazol­ato)-μ_2_-formato]

**DOI:** 10.1107/S160053680802299X

**Published:** 2008-07-26

**Authors:** Ya-Wen Zhang, Gong Zhang, Yan-Yan Sun, Lin Cheng

**Affiliations:** a Department of Chemistry and Chemical Engineering, Southeast University, Nanjing, People’s Republic of China; b Department of Chemistry and Chemical Engineering, State Key Laboratory of Coordination Chemistry, Nanjing University, Nanjing, People’s Republic of China

## Abstract

Owing to the presence of crystallographic twofold rotation axes (site symmetry 2, Wyckoff letters *e* and *f*), the asymmetric unit of the title compound, [Mn(C_12_H_8_N_5_)(CHO_2_)]_*n*_, contains one-half of an Mn^II^ cation, one-half of a bpt anion (Hbpt is 3,5-di-2-pyridyl-4*H*-1,2,4-triazole) and one-half of a formate anion. The bpt and formate ligands occupy the same *C*_2_ symmetry, while the Mn^II^ ion resides on another crystallographic twofold rotation axis. Each bpt ligand acts as a *cis*-bis-chelate to ligate two Mn^II^ ions into a one-dimensional chain running along the crystallographic 4_1_ screw axis. Adjacent Mn^II^ ions are further bridged by a μ_2_-formate ligand, completing the distorted octa­hedral coordination geometry of the cation.

## Related literature

For related literature, see: Zhang (2005 ▶); Chen & Tong (2007 ▶). For related structures, see: Cheng *et al.* (2007*a* ▶,*b* ▶).
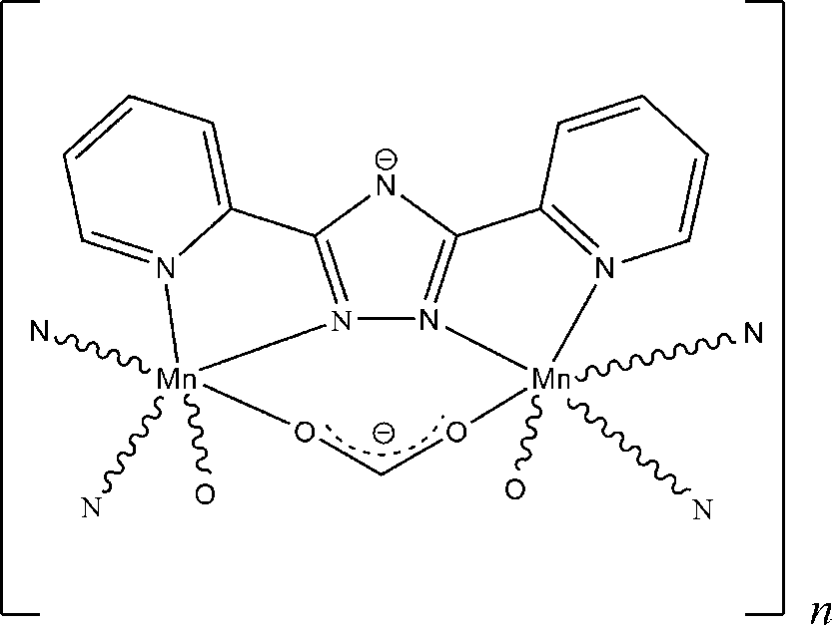


## Experimental

### 

#### Crystal data

[Mn(C_12_H_8_N_5_)(CHO_2_)]
*M* *_r_* = 322.20
Tetragonal, 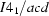

*a* = 19.124 (5) Å
*c* = 14.9120 (4) Å
*V* = 5454 (2) Å^3^
*Z* = 16
Mo *K*α radiation
μ = 0.98 mm^−1^
*T* = 293 (2) K
0.15 × 0.09 × 0.06 mm

#### Data collection

Bruker APEX CCD diffractometer
Absorption correction: multi-scan (*SADABS*; Sheldrick, 2000 ▶) *T*_min_ = 0.867, *T*_max_ = 0.944
14412 measured reflections
1346 independent reflections
1225 reflections with *I* > 2σ(*I*)
*R*_int_ = 0.054

#### Refinement

*R*[*F* ^2^ > 2σ(*F* ^2^)] = 0.055
*wR*(*F* ^2^) = 0.121
*S* = 1.09
1346 reflections
97 parameters
H-atom parameters constrained
Δρ_max_ = 0.39 e Å^−3^
Δρ_min_ = −0.37 e Å^−3^

### 

Data collection: *SMART* (Bruker, 2000 ▶); cell refinement: *SAINT* (Bruker, 2000 ▶); data reduction: *SAINT*; program(s) used to solve structure: *SHELXS97* (Sheldrick, 2008 ▶); program(s) used to refine structure: *SHELXL97* (Sheldrick, 2008 ▶); molecular graphics: *SHELXTL* (Sheldrick, 2008 ▶); software used to prepare material for publication: *SHELXTL*.

## Supplementary Material

Crystal structure: contains datablocks I, global. DOI: 10.1107/S160053680802299X/si2099sup1.cif

Structure factors: contains datablocks I. DOI: 10.1107/S160053680802299X/si2099Isup2.hkl

Additional supplementary materials:  crystallographic information; 3D view; checkCIF report

## Figures and Tables

**Fig. 1.**
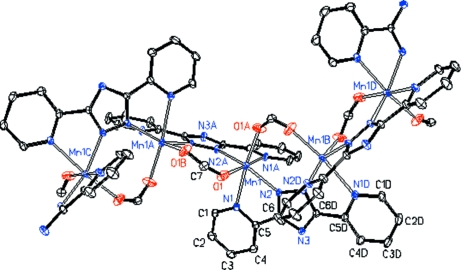
The one-dimensional chain of the title compound with 30% thermal ellipsoids. All the hydrogen atoms are omitted for clarity. Symmetry codes: a: 5/4 - x, -1/4 - y, 1/4 - z; b: 3/2 - x, y, 1/2 - z; c: 1/4 + x, -1/4 - y, -1/4 + z; d: x, -y, 1/2 - z.

## supplementary crystallographic information

### Comment

Recently, solvothermal *in situ* ligand reactions have been a rapidly growing field concerning with the formation of *in situ* generated mixed-ligand coordination polymers that can not be easily obtained: a. one-pot synthesis of some unusual organic ligands that are inaccessible or not easily obtainable *via* conventional methods, and b. which are very promising as a bridge between coordination and synthetic organic chemistry (Zhang, 2005; Chen & Tong, 2007). During our research of the reaction mechanisms of different organonitriles with hydrazine hydrate (Cheng *et al.*, 2007*a*,*b*), a new one-dimensional mixed-ligand polymeric manganese(II) complex, [Mn(bpt)0.5(HCOO)0.5]*n* (Hbpt = 3,5-bis(2-pyridyl)-4*H*-1,2,4-triazole) has been synthesized and characterized by single-crystal X-ray diffraction.

The asymmetric unit of the title compound contains half a Mn^II^ cation, half a bpt and half a formato anion. In the compound, the Mn^II^ ion lies on a twofold rotation axis, at position (*x*, 1/4 + *x*, 1/8), Wyckoff letter f. Neighboring twofold rotation axes in the high symmetric space group I 4_1_/a 2/c 2/d, running through atoms C7, H7A in the formato anion and through atom N3 of the triazole group, at positions (3/4, 3/4 + *x*, 0) and (*x*, 0, 1/4), respectively, both with Wyckoff letter e. The Mn^II^ ion displays a slightly distorted octahedral geometry, being surrounded by two chelating bpt ligands and two oxygen atoms from two µ_2_-formato ligands, linking the half molecules in the complex to a one-dimensional chain extending along the crystallographic 4_1_-screw axis. The shortest Mn···Mn distance in the chain is 4.366 (5) Å.

### Experimental

A mixture of 4-cyanopyridine (0.416 g, 4.0 mmol), 80% hydrazine hydrate (2 ml), Mn(HCOO)_2_.2H_2_O (0.181 g, 1 mmol) and DMF (6 ml) was heated in a 15-ml Teflon-lined autoclave at 180° for 3 days, followed by slow cooling (5° h-1) to room temperature. The resulting mixture was washed with water, and pale-yellow block crystals were collected and dried in air [yield 1.0% (3.2 mg) based on Mn^II^].

### Refinement

H atoms were positioned geometrically and refined using a riding model with constraint distances C—H = 0.93 Å and with *U*_iso_(H) = 1.2*U*_eq_(C).

### Crystal data

**Table:**

[Mn(C_12_H_8_N_5_)(CHO_2_)]	*Z* = 16
*M**_r_* = 322.20	*F*_000_ = 2608
Tetragonal, *I*4_1_/*a**c**d*	*D*_x_ = 1.570 Mg m^−^^3^
Hall symbol: -I 4bd 2c	Mo *K*α radiation λ = 0.71073 Å
*a* = 19.124 (5) Å	Cell parameters from 810 reflections
*b* = 19.124 (5) Å	θ = 2.5–28.0º
*c* = 14.9120 (4) Å	µ = 0.98 mm^−^^1^
α = 90º	*T* = 293 (2) K
β = 90º	Needle-like, yellow
γ = 90º	0.15 × 0.09 × 0.06 mm
*V* = 5454 (2) Å^3^	

### Data collection

**Table:**

Bruker APEX CCD diffractometer	1346 independent reflections
Radiation source: fine-focus sealed tube	1225 reflections with *I* > 2σ(*I*)
Monochromator: graphite	*R*_int_ = 0.054
*T* = 293(2) K	θ_max_ = 26.0º
φ and ω scans	θ_min_ = 2.1º
Absorption correction: multi-scan(SADABS; Sheldrick, 2000)	*h* = −23→18
*T*_min_ = 0.867, *T*_max_ = 0.944	*k* = −23→23
14412 measured reflections	*l* = −18→17

### Refinement

**Table:**

Refinement on *F*^2^	Secondary atom site location: difference Fourier map
Least-squares matrix: full	Hydrogen site location: inferred from neighbouring sites
*R*[*F*^2^ > 2σ(*F*^2^)] = 0.055	H-atom parameters constrained
*wR*(*F*^2^) = 0.121	*w* = 1/[σ^2^(*F*_o_^2^) + (0.0491*P*)^2^ + 25.4234*P*] where *P* = (*F*_o_^2^ + 2*F*_c_^2^)/3
*S* = 1.09	(Δ/σ)_max_ < 0.001
1346 reflections	Δρ_max_ = 0.39 e Å^−^^3^
97 parameters	Δρ_min_ = −0.37 e Å^−^^3^
Primary atom site location: structure-invariant direct methods	Extinction correction: none

### Special details

**Table:**

Geometry. All e.s.d.'s (except the e.s.d. in the dihedral angle between two l.s. planes) are estimated using the full covariance matrix. The cell e.s.d.'s are taken into account individually in the estimation of e.s.d.'s in distances, angles and torsion angles; correlations between e.s.d.'s in cell parameters are only used when they are defined by crystal symmetry. An approximate (isotropic) treatment of cell e.s.d.'s is used for estimating e.s.d.'s involving l.s. planes.
Refinement. Refinement of *F*^2^ against ALL reflections. The weighted *R*-factor *wR* and goodness of fit *S* are based on *F*^2^, conventional *R*-factors *R* are based on *F*, with *F* set to zero for negative *F*^2^. The threshold expression of *F*^2^ > σ(*F*^2^) is used only for calculating *R*-factors(gt) *etc*. and is not relevant to the choice of reflections for refinement. *R*-factors based on *F*^2^ are statistically about twice as large as those based on *F*, and *R*- factors based on ALL data will be even larger.

### Fractional atomic coordinates and isotropic or equivalent isotropic displacement parameters (Å^2^)

**Table:**

	*x*	*y*	*z*	*U*_iso_*/*U*_eq_	
Mn1	0.69057 (3)	−0.05943 (3)	0.1250	0.0215 (2)	
C1	0.56957 (19)	−0.11100 (19)	−0.0239 (2)	0.0318 (8)	
H1A	0.6082	−0.1244	−0.0576	0.038*	
C2	0.5042 (2)	−0.1237 (2)	−0.0580 (2)	0.0402 (10)	
H2A	0.4989	−0.1454	−0.1134	0.048*	
C3	0.4464 (2)	−0.1038 (2)	−0.0088 (3)	0.0431 (10)	
H3A	0.4015	−0.1118	−0.0305	0.052*	
C4	0.45662 (19)	−0.0717 (2)	0.0735 (2)	0.0358 (9)	
H4A	0.4187	−0.0579	0.1082	0.043*	
C5	0.52424 (17)	−0.06066 (18)	0.1029 (2)	0.0259 (7)	
C6	0.54152 (16)	−0.02572 (17)	0.1886 (2)	0.0214 (7)	
C7	0.7500	0.0594 (3)	0.0000	0.0300 (11)	
H7A	0.7500	0.1080	0.0000	0.036*	
N1	0.58042 (15)	−0.08037 (14)	0.05536 (18)	0.0250 (6)	
N2	0.60816 (13)	−0.01663 (13)	0.20974 (16)	0.0196 (6)	
N3	0.4964 (2)	0.0000	0.2500	0.0272 (9)	
O1	0.70537 (14)	0.03180 (15)	0.0481 (2)	0.0501 (8)	

### Atomic displacement parameters (Å^2^)

**Table:**

	*U*^11^	*U*^22^	*U*^33^	*U*^12^	*U*^13^	*U*^23^
Mn1	0.0221 (3)	0.0221 (3)	0.0203 (4)	0.0017 (3)	0.00432 (19)	−0.00432 (19)
C1	0.0325 (19)	0.041 (2)	0.0219 (17)	0.0094 (16)	−0.0010 (15)	−0.0133 (15)
C2	0.041 (2)	0.051 (2)	0.0280 (18)	0.0110 (19)	−0.0106 (17)	−0.0199 (18)
C3	0.033 (2)	0.060 (3)	0.037 (2)	0.0061 (18)	−0.0149 (17)	−0.019 (2)
C4	0.0266 (19)	0.048 (2)	0.0325 (19)	0.0032 (16)	−0.0025 (16)	−0.0150 (17)
C5	0.0272 (18)	0.0303 (18)	0.0202 (16)	0.0008 (14)	−0.0028 (13)	−0.0057 (14)
C6	0.0210 (16)	0.0264 (17)	0.0167 (14)	0.0000 (13)	−0.0021 (12)	−0.0049 (13)
C7	0.032 (3)	0.023 (2)	0.035 (3)	0.000	0.001 (2)	0.000
N1	0.0279 (15)	0.0275 (15)	0.0197 (14)	0.0018 (12)	−0.0009 (11)	−0.0086 (11)
N2	0.0225 (14)	0.0210 (14)	0.0154 (12)	−0.0015 (10)	−0.0012 (11)	−0.0058 (10)
N3	0.0205 (19)	0.039 (2)	0.0217 (18)	0.000	0.000	−0.0090 (17)
O1	0.0370 (16)	0.0438 (16)	0.069 (2)	0.0047 (13)	0.0165 (15)	0.0253 (15)

### Geometric parameters (Å, °)

**Table:**

Mn1—O1	2.107 (3)	C3—H3A	0.9300
Mn1—O1^i^	2.107 (3)	C4—C5	1.382 (5)
Mn1—N2^i^	2.180 (3)	C4—H4A	0.9300
Mn1—N2	2.180 (3)	C5—N1	1.341 (4)
Mn1—N1	2.382 (3)	C5—C6	1.480 (4)
Mn1—N1^i^	2.382 (3)	C6—N2	1.324 (4)
C1—N1	1.335 (4)	C6—N3	1.350 (4)
C1—C2	1.372 (5)	C7—O1	1.234 (3)
C1—H1A	0.9300	C7—O1^ii^	1.234 (3)
C2—C3	1.379 (5)	C7—H7A	0.9300
C2—H2A	0.9300	N2—N2^iii^	1.359 (5)
C3—C4	1.386 (5)	N3—C6^iii^	1.350 (4)
			
O1—Mn1—O1^i^	94.23 (17)	C2—C3—H3A	120.6
O1—Mn1—N2^i^	103.46 (11)	C4—C3—H3A	120.6
O1^i^—Mn1—N2^i^	95.84 (10)	C5—C4—C3	118.7 (3)
O1—Mn1—N2	95.84 (10)	C5—C4—H4A	120.6
O1^i^—Mn1—N2	103.46 (11)	C3—C4—H4A	120.6
N2^i^—Mn1—N2	151.55 (13)	N1—C5—C4	122.6 (3)
O1—Mn1—N1	91.19 (11)	N1—C5—C6	113.9 (3)
O1^i^—Mn1—N1	172.74 (12)	C4—C5—C6	123.5 (3)
N2^i^—Mn1—N1	87.58 (9)	N2—C6—N3	114.0 (3)
N2—Mn1—N1	71.13 (9)	N2—C6—C5	118.6 (3)
O1—Mn1—N1^i^	172.74 (12)	N3—C6—C5	127.4 (3)
O1^i^—Mn1—N1^i^	91.19 (11)	O1—C7—O1^ii^	129.3 (5)
N2^i^—Mn1—N1^i^	71.13 (9)	O1—C7—H7A	115.3
N2—Mn1—N1^i^	87.58 (9)	O1^ii^—C7—H7A	115.3
N1—Mn1—N1^i^	83.84 (14)	C1—N1—C5	117.8 (3)
N1—C1—C2	123.2 (3)	C1—N1—Mn1	126.6 (2)
N1—C1—H1A	118.4	C5—N1—Mn1	115.5 (2)
C2—C1—H1A	118.4	C6—N2—N2^iii^	105.75 (17)
C1—C2—C3	119.0 (3)	C6—N2—Mn1	120.59 (19)
C1—C2—H2A	120.5	N2^iii^—N2—Mn1	133.48 (7)
C3—C2—H2A	120.5	C6—N3—C6^iii^	100.6 (4)
C2—C3—C4	118.7 (4)	C7—O1—Mn1	139.8 (3)

Symmetry codes: (i) *y*+3/4, *x*−3/4, −*z*+1/4; (ii) −*x*+3/2, *y*, −*z*; (iii) *x*, −*y*, −*z*+1/2.

## References

[bb1] Bruker (2000). *SMART* and *SAINT* Bruker AXS Inc., Madison, Wisconsin, USA.

[bb2] Chen, X.-M. & Tong, M.-L. (2007). *Acc. Chem. Res.* **40**, 162–170. 10.1021/ar068084p 17309196

[bb3] Cheng, L., Zhang, W.-X., Ye, B.-H., Lin, J.-B. & Chen, X.-M. (2007*a*). *Inorg. Chem.* **46**, 1135–1143. 10.1021/ic061303i 17291112

[bb4] Cheng, L., Zhang, W.-X., Ye, B.-H., Lin, J.-B. & Chen, X.-M. (2007*b*). *Eur. J. Inorg. Chem.* pp. 2668–2676.

[bb5] Sheldrick, G. M. (2000). *SADABS* University of Göttingen, Germany.

[bb6] Sheldrick, G. M. (2008). *Acta Cryst.* A**64**, 112–122. 10.1107/S0108767307043930 18156677

[bb7] Zhang, X.-M. (2005). *Coord. Chem. Rev.* **249**, 1201–1219.

